# Balloon flight test of a CeBr_3_ detector with silicon photomultiplier readout

**DOI:** 10.1007/s10686-021-09767-z

**Published:** 2021-06-25

**Authors:** David Murphy, Joseph Mangan, Alexei Ulyanov, Sarah Walsh, Rachel Dunwoody, Lorraine Hanlon, Brian Shortt, Sheila McBreen

**Affiliations:** 1 School of Physics and Centre for Space Research, University College Dublin, Dublin 4, Ireland; 2 European Space Agency, ESTEC, 2200 AG Noordwijk, The Netherlands

**Keywords:** Gamma-ray detector, Balloon

## Abstract

Recent advances in silicon photomultiplier (SiPM) technology and new scintillator materials allow for the creation of compact high-performance gamma-ray detectors which can be deployed on small low-cost satellites. A small number of such satellites can provide full sky coverage and complement, or in some cases replace the existing gamma-ray missions in detection of transient gamma-ray events. The aim of this study is to test gamma-ray detection using a novel commercially available CeBr_3_ scintillator combined with SiPM readout in a near-space environment and inform further technology development for a future space mission. A prototype gamma-ray detector was built using a CeBr_3_ scintillator and an array of 16 J-Series SiPMs by ON Semiconductor. SiPM readout was performed using SIPHRA, a radiation-tolerant low-power integrated circuit developed by IDEAS. The detector was flown as a piggyback payload on the Advanced Scintillator Compton Telescope balloon flight from Columbia Scientific Balloon Facility. The payload included the detector, a Raspberry Pi on-board computer, a custom power supply board, temperature and pressure sensors, a Global Navigation Satellite System receiver and a satellite modem. The balloon delivered the detector to 37 km altitude where its detection capabilities and readout were tested in the radiation-intense near-space environment. The detector demonstrated continuous operation during the 8-hour flight and after the landing. It performed spectral measurements in an energy range of 100 keV to 8 MeV and observed the 511 keV gamma-ray line arising from positron annihilation in the atmosphere with full width half maximum of 6.8%. During ascent and descent, the detector count rate peaked at an altitude of 16 km corresponding to the point of maximum radiation intensity in the atmosphere. Despite several engineering issues discovered after the flight test, the results of this study confirm the feasibility of using CeBr_3_ scintillator, SiPMs, and SIPHRA in future space missions.

## Introduction

Gamma-ray space missions including the Neil Gehrels Swift Observatory [12], Fermi Gamma-ray Space Telescope [7, 17], and INTEGRAL [37] observed and discovered many new sources, including transient sources such as gamma-ray bursts (GRBs; e.g. [34]), soft gamma-ray repeaters (e.g. [29]), and terrestrial gamma flashes (e.g. [8]). The need for gamma-ray telescopes was recently heightened with the beginning of the gravitational wave era in 2015 with the direct detection of gravitational waves from a binary black hole (BBH) merger by [2], followed by the detection of additional nine BBH mergers [1]. The detection of the gravitational wave signal from a binary neutron star merger, GW170817 [3] by the LIGO [14, 16] and Virgo [5] experiments in coincidence with a short gamma-ray burst, GRB 170817A [13] was another breakthrough discovery which led to a large follow-up campaign by space and ground based telescopes and highlighted the importance of gamma-ray missions to the future detection and localisation of GRBs in the context of multi-messenger astronomy.

A challenge for future gamma-ray astronomy is the potential lack of future missions for transient follow-up. All current gamma-ray missions have already exceeded the nominal mission lifetimes. Future large missions include SVOM which is a mission with limited sky coverage due for launch in 2021 [10]. While other mission proposals are under review, e.g. THESEUS [6] and AMEGO [9], no follow-on mission is firmly in the pipeline from any of the large space agencies opening up a gap in capabilities in the coming years. This coincides with the timeline for major upgrades of the gravitational wave facilities [4] which will likely lead to the discovery of more gravitational wave sources including binary neutron star and black hole-neutron star mergers, generally believed to be the progenitors of short GRBs. The simultaneous discovery of gravitational wave and electromagnetic signatures requires dedicated and coordinated observations by large communities of both ground and space-based observatories.

Even if the current missions were to remain operational, they do not provide full sky coverage [23]. One way to improve coverage at low cost is via CubeSats with instrumentation capable of detecting gamma rays, such as BurstCube [23], MoonBEAM [15], GRID [36], and HERMES [11]. The requirements to adequately detect and localise GRBs in the multimessenger era can be achieved with the existing fleet of GRB detecting instruments by support from such CubeSat programs.

All the mentioned CubeSat missions employ either silicon photomultipliers (SiPMs) or silicon drift detectors (SDDs) for detection of scintillation light produced by gamma rays. Although SiPMs have replaced traditional photomultiplier tubes (PMTs) in many terrestrial applications, this technology is still very new in the space industry. Several small missions have been designed to space-qualify SiPMs together with new scintillator materials, including the Strontium Iodide Radiation Instrument (SIRI) launched in December 2018 [19] which used a europium-doped strontium iodide (SrI_2_:Eu) scintillator, and the Gamma-ray Module (GMOD) to be flown on board the 2U EIRSAT-1 CubeSat [20]. Based on previous developments using novel lanthanum bromide (LaBr_3_) and cerium bromide (CeBr_3_) scintillators [30, 31], GMOD will comprise a 25mm×25mm×40mm CeBr_3_ crystal and an array of 16 J-Series SiPMs by ON Semiconductor (formerly SensL). The readout will be performed using Silicon Photomultiplier Readout ASIC, a low-power radiation-tolerant application specific integrated circuit (ASIC) recently developed by Integrated Detector Electronics AS (IDEAS), Norway [18, 32].

This paper presents the Gamma-ray Module Demonstrator (GMoDem), a self-contained development model of GMOD which was flown on a high-altitude balloon flight in order to test in a near-space environment the technologies that will be used in the GMOD detector. GMoDem was hosted as a piggyback payload on Advanced Scintillator Compton Telescope (ASCOT) balloon flight [27] launched from Columbia Scientific Balloon Facility (CSBF) in Texas. The paper describes the payload design, flight preparations, data analysis and reports the results of the flight test.

## GMoDem hardware

**Fig. 1:**
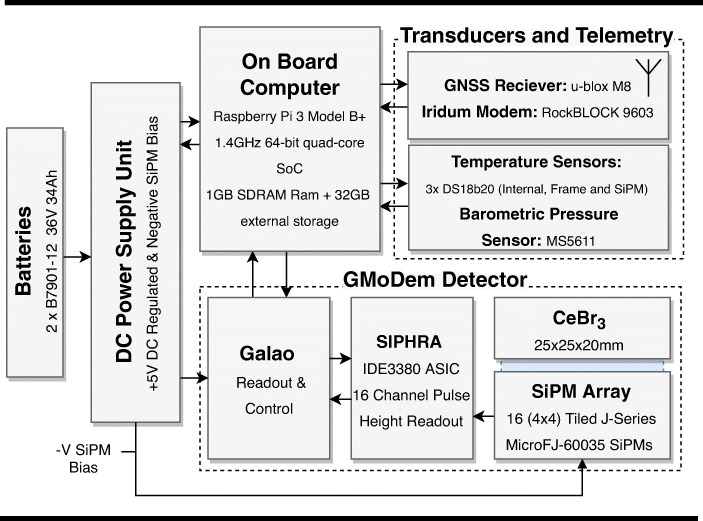
Overview of the GMoDem payload components

The components being evaluated in GMoDem are those which will comprise the GMOD detector assembly: a CeBr_3_ scintillator coupled to an SiPM array with readout via the SIPHRA ASIC. For the purposes of operating these components in GMoDem, a SIPHRA evaluation board is used, connected to an IDEAS Galao board designed for evaluation of IDEAS readout ASICs. The Galao board is in turn connected via ethernet to a single-board computer (SBC) which processes and records events from the detector. Figure 1 gives an overview of the GMoDem components which are described in detail below.

### GMoDem detector assembly

**Fig. 2:**
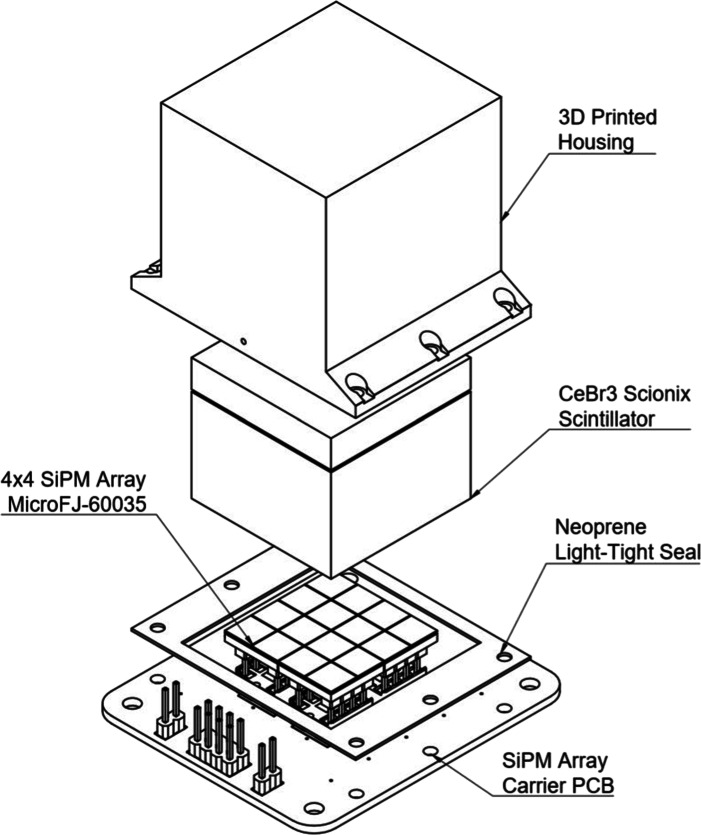
GMoDem detector comprising the CeBr_3_ scintillator, SiPM array and light-tight enclosure

The GMoDem detector assembly (Fig. 2) comprises a 25 mm × 25 mm × 20 mm CeBr_3_ scintillator crystal coupled to an array of sixteen ON Semiconductor J-Series MicroFJ-60035 SiPMs. As CeBr_3_ is hygroscopic, the crystal was supplied by SCIONIX in a hermetically sealed aluminium enclosure with a quartz window exposing one of the 25 mm × 25 mm faces. Optical gel was applied to the quartz window to improve light transmission to the SiPMs.

The SiPM array is formed using four 2 × 2 SiPM arrays (ON Semiconductor ArrayJ-60035-4P-PCB). This configuration is informed by the SIPHRA readout requirements and the short development time available for GMoDem. The SIPHRA current mode input stage (CMIS) requires negative charge input which therefore requires a connection to the SiPM cathode. Ideally, a custom 16-SiPM common anode array such as that planned for GMOD would have been used, however the individual J-series SiPMs are provided in Through Silicon Via (TSV) packages which would have required an outsourced soldering process which could not be completed in the available time frame. J-series 6 mm tileable SiPMs with factory-attached connectors were sought instead but these are unfortunately only available as 2 × 2 or 4 × 4 common cathode arrays. While these arrays can be used with SIPHRA, only a single input channel can be used for the entire array rather than one input per SiPM. A compromise of using as many of SIPHRA’s input channels as possible and using commercially available socketed SiPMs led to this configuration of a 4 × 4 SiPM array which was actually a 2 × 2 common-anode array of 2 × 2 common-cathode arrays.

A carrier printed circuit board (PCB) was designed and assembled in-house to accommodate the four ON Semiconductor arrays using suitable sockets. The sockets were positioned to provide the correct 0.2 mm spacing between the arrays, matching the SiPM spacing used within the arrays, giving a uniform spacing between all sixteen SiPMs. The carrier PCB also featured the power and readout connectors, four low-pass filters to filter the bias supply to each of the common-anode arrays, and a series of mounting holes used to attach a light-tight shroud over the assembly and to mount the assembly within GMoDem. The light-tight shroud was 3D-printed using a design that had previously performed well in the laboratory environment and was attached to the carrier PCB using a neoprene gasket to maintain a light-tight seal.

The SiPM array was operated at a fixed overvoltage above the SiPM breakdown voltage to maintain a constant gain. As the breakdown voltage is temperature dependent, a DS18b20 temperature sensor was attached to the underside of the carrier PCB to allow the flight computer to determine the array temperature and apply the appropriate bias voltage using the programmable PSU (see Section 2.5).

### Silicon photomultiplier readout

The SIPHRA ASIC was designed specifically for the purpose of readout and digitisation of SiPM sensors in space applications. It was therefore designed with latch-up immunity, single event upset mitigation and error correction, and low-power considerations [18]. As SIPHRA will be used in GMOD, it is one of the main components being evaluated in GMoDem.

SIPHRA has sixteen SiPM input channels and an internal seventeenth summing channel which provides a sum of the sixteen inputs. As described in Section 2.1, only four of the sixteen input channels are used in GMoDem.

SIPHRA is highly configurable via a Serial Peripheral Interface (SPI) and in GMoDem it is used as a pulse height spectrometer. Each of the four SiPM outputs is connected to a CMIS which provides programmable input attenuation. Each CMIS is connected to an independent current integrator, pulse shaper, and track and hold unit. The current integrator, pulse shaper, and track and hold unit chain is also replicated for the summing channel whose current integrator is connected to all sixteen CMISs. The track and hold units are connected to a single 12-bit analogue to digital converter (ADC) via an analog multiplexer. The digitised signals are clocked out over a serial output which is synchronised to SIPHRA’s input clock source.

The SIPHRA used in GMoDem was a chip on board (CoB) version featuring a silicon die wire-bonded directly to a large 129 mm × 124 mm evaluation PCB with 0.1-inch pin-headers and SMA sockets for connection to the SiPMs and with a large board-to-board connector to connect to the IDEAS Galao evaluation platform. An aluminium chip cover is bolted to the PCB to protect the SIPHRA silicon die.

### Data processing and storage

A Raspberry Pi 3 Model B+ SBC is used for the on-board computer (OBC). The interface between the OBC and SIPHRA is via the Galao board which is connected to the OBC using ethernet. Configuration of SIPHRA is performed over SPI by Galao in response to commands from the OBC. Event data generated by SIPHRA are transmitted on a serial data line to Galao and then relayed to the OBC.

A 32 GB industrial grade SD card was loaded with a Linux operating system and a series of Python scripts which comprised the flight software. This SD card was also used as a backup storage location for experiment data. The primary storage for the experiment data was a rugged USB flash drive which was connected to the OBC via a short USB extension cable and mounted inside the GMoDem enclosure in a location which was easily accessible to the balloon recovery team in the event that not all of the experiment could be recovered.

The flight software was written to be completely autonomous requiring simply that power be connected to GMoDem and the Remove Before Flight (RBF) pin removed for data acquisition to begin. A process control system was used to ensure that any processes which ended during the flight were automatically restarted.

In addition to automatic configuration and data acquisition from SIPHRA, the flight software logged housekeeping data from a number of sources connected to the OBC. Three DS18b20 ‘1-wire’ temperature sensors were used to monitor the internal temperature inside the GMoDem enclosure, the external temperature, and the temperature of the SiPM array. An MS5611 I^2^C barometric pressure sensor was used to monitor pressure. A u-blox M8 Global Navigation Satellite System (GNSS) receiver was used to give positional data. The u-blox receiver was chosen because it can easily be configured in ‘airborne’ mode which will continue to give valid position data at altitudes up to 50 km while many other brands of GNSS receiver will deactivate at high altitudes in an excessively cautious effort to comply with International Traffic in Arms Regulations. The temperatures, pressure, latitude, longitude, and altitude were recorded every 10 s as housekeeping data throughout the flight. As with the experiment data, the housekeeping data was also stored on both the SD card and USB flash drive.

### Communication

As GMoDem was a piggyback payload, the communications systems provided by National Aeronautics and Space Administration (NASA) were not used in order to have as little an impact as possible on the operations of the ASCOT host. Instead, GMoDem included a RockBLOCK 9603 transceiver based on the Iridium 9603 modem allowing for Short Burst Data (SBD) satellite communication which was advantageous as it did not require any local ground infrastructure. The transceiver was connected to the on-board computer using a USB to serial adapter and the flight software controlled the modem using a simple AT command interface.

Iridium SBD is analogous to SMS on cell phones, it is not capable of transferring a lot of data such as experiment data, but was useful for monitoring of housekeeping data. The flight software was programmed to attempt to transmit housekeeping data every 5 minutes throughout the flight. During these transmissions, messages from the ground could also be downloaded from the satellite. The flight software had a small command set that could be parsed from incoming messages to modify parameters or manually reset various flight software components.

The network access provider, RockBLOCK, provided a web interface to send messages to and display messages from GMoDem. Messages were also relayed to the ground software, then parsed and displayed in a dashboard application on http://gmodem.spacescience.ie.

### Power

A custom PSU was designed and built for GMoDem to power the various components and included an RBF pin, software controlled voltage regulation of the SiPM bias voltage, and the ability to power-cycle the detector from the on-board computer.

GMoDem was powered by two B7901-12 batteries which were supplied by CSBF and were mounted beside GMoDem on the gondola. Each battery contained twelve G62 lithium sulfur dioxide (LiSO_2_) cells in series providing a nominal unloaded voltage of 36 V and capacity of 34 Ah. The batteries were connected in parallel using a wiring harness and then connected to the GMoDem PSU to extend the available running time of the experiment. The power input to GMoDem from the batteries was via a Neutrik twist-lock-latching powerCON connector, which was chosen to provide a very easy way for CSBF technicians to disconnect power to the experiment during recovery.

The PSU took advantage of having such a high voltage available from the batteries to bias the SiPMs directly without having to use a boost converter. The positive terminal of the battery was used as GMoDem’s ground reference, allowing the negative terminal to be used as a − 36 V rail. The battery was also connected to a JTD2048S05 isolated 5 V voltage regulator. The negative output of the regulator is connected to GMoDem’s ground, creating 5 V, GND and − 36 V rails. The 5 V is used to power the on-board computer (and USB connected components) and Galao. SIPHRA was powered from the voltage and current sources included on the Galao board. An inhibit on the 5 V voltage regulator is used as an RBF pin switch, powering down the on-board computer, Galao and SIPHRA when the pin is present, providing a very easy way to power on and off GMoDem.

As the SiPM gain is related to the overvoltage, the voltage at which the SiPM is biased above the breakdown voltage, the detector gain can be adjusted by adjusting the SiPM bias voltage. Conversely, maintaining a constant gain requires maintaining a constant overvoltage. The J-series SiPMs offer good temperature stability but the breakdown voltage does have a temperature dependence of 21.5 mV/°C [22]. To maintain a constant gain, the bias voltage needs to be continuously adjusted to compensate for variations of the SiPM temperature.

A series of voltage references and high voltage, high current OP551 op-amps were used to regulate the − 36 V battery voltage to the desired SiPM bias voltage. The first stage op-amp in an inverting configuration generated a − 15 V output from a 2.5 V reference. This output and the 0–5 V output from an SPI digital to analogue converter (DAC) connected to the on-board computer were used in a second stage inverting op-amp with a gain of 1.75 to generate an SiPM bias voltage range of between − 26.25 V and − 30 V which could be set by the flight software.

Galao and SIPHRA were powered through a solid state relay which was controlled by a general purpose input/output (GPIO) pin on the on-board computer. This allowed the detector to be power-cycled by telecommand or automatically by the flight software in response to communication errors between the on-board computer and Galao.

### Structure / enclosure

**Fig. 3:**
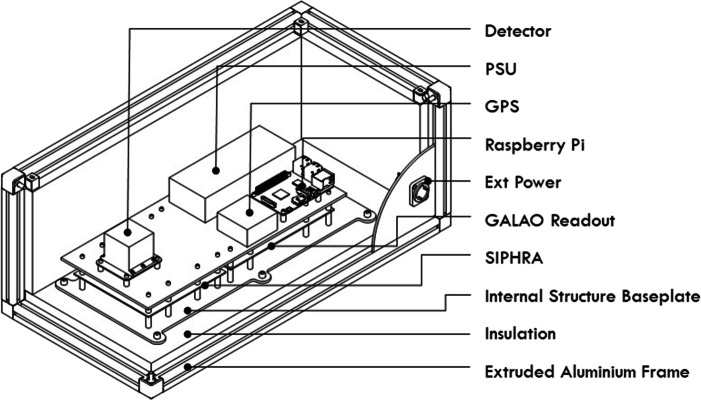
Mechanical model of the GMoDem experiment comprising two plates where the components are mounted. The detector, power, storage, and GPS are mounted to the upper plate and the readout solution on the lower plate

**Fig. 4:**
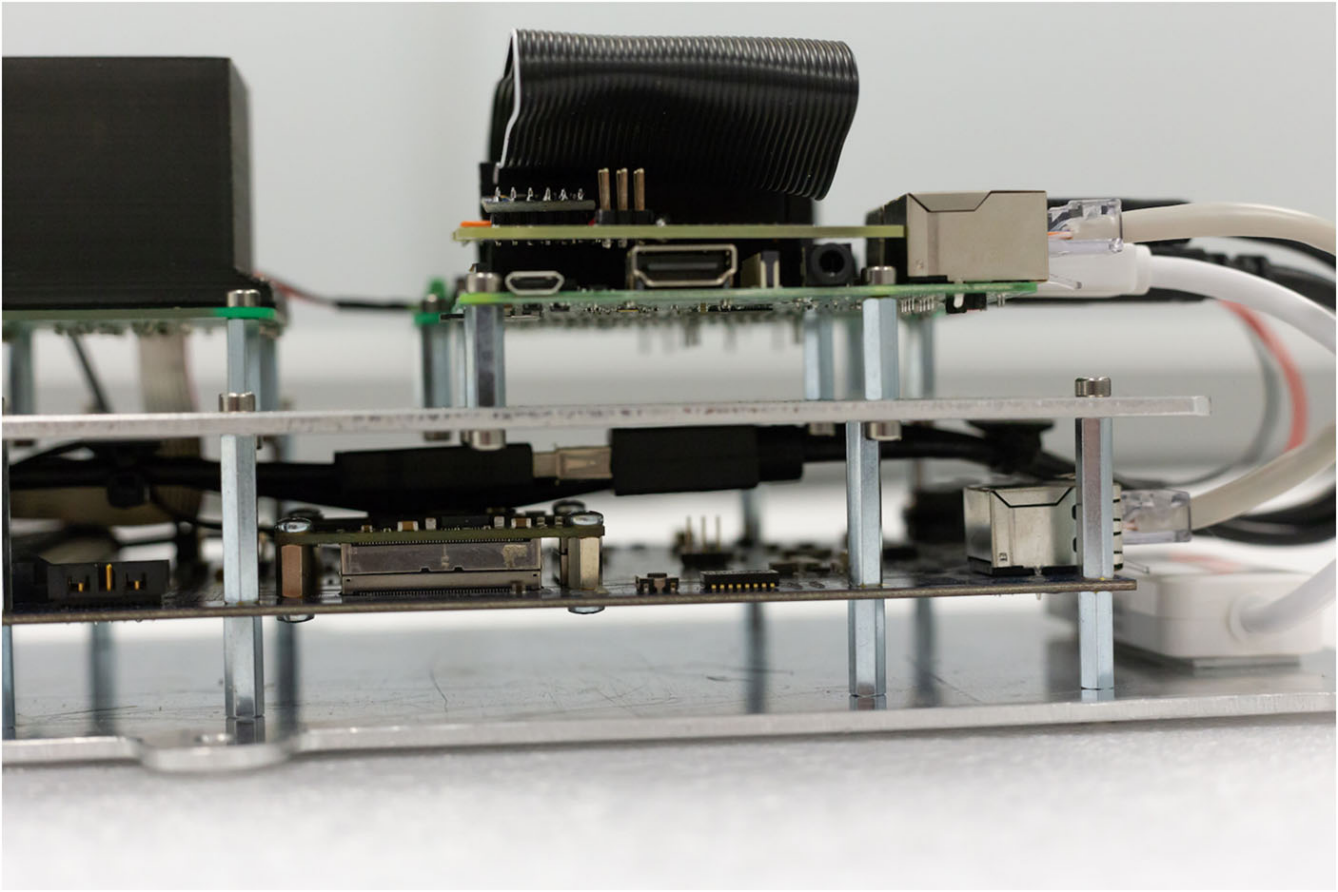
The internal GMoDem structure of aluminium plates and components

The internal structure (Fig. 3) consisted of a stack of components mounted on two custom aluminium plates. The overall size of the plates were dictated by the size of the Galao and SIPHRA boards which were mounted securely between the two plates using M3 stand-offs through all available mounting holes on the PCBs. The detector assembly and data handling components were mounted to the top plate, typically via M3 stand-offs and screws though in some cases the components had no mounting holes and so friction-fit brackets were 3D printed and secured to the aluminium plate with adhesive tape. This stack arrangement is shown in Fig. 4. The bottom plate included six mounting tabs which were used to bolt the assembly down onto M5 stand-offs on the bottom of the enclosure. The tabs extended out beyond the top layer so that the entire assembly could be easily removed and reinstalled in the enclosure quickly.

The enclosure was designed to be mounted to the ASCOT gondola as quickly and easily as possible and so was manufactured from 20 mm extruded aluminium profile with plastic panels mounted into the internal slots and secured with a rubber locking strip. The insides of the plastic panels were insulated with 25 mm of extruded polystyrene insulation. With this construction, the edges of all faces of the enclosure had continuous T-slots into which T-slot nuts could be inserted and brackets attached, allowing a very flexible mounting solution.

**Fig. 5:**
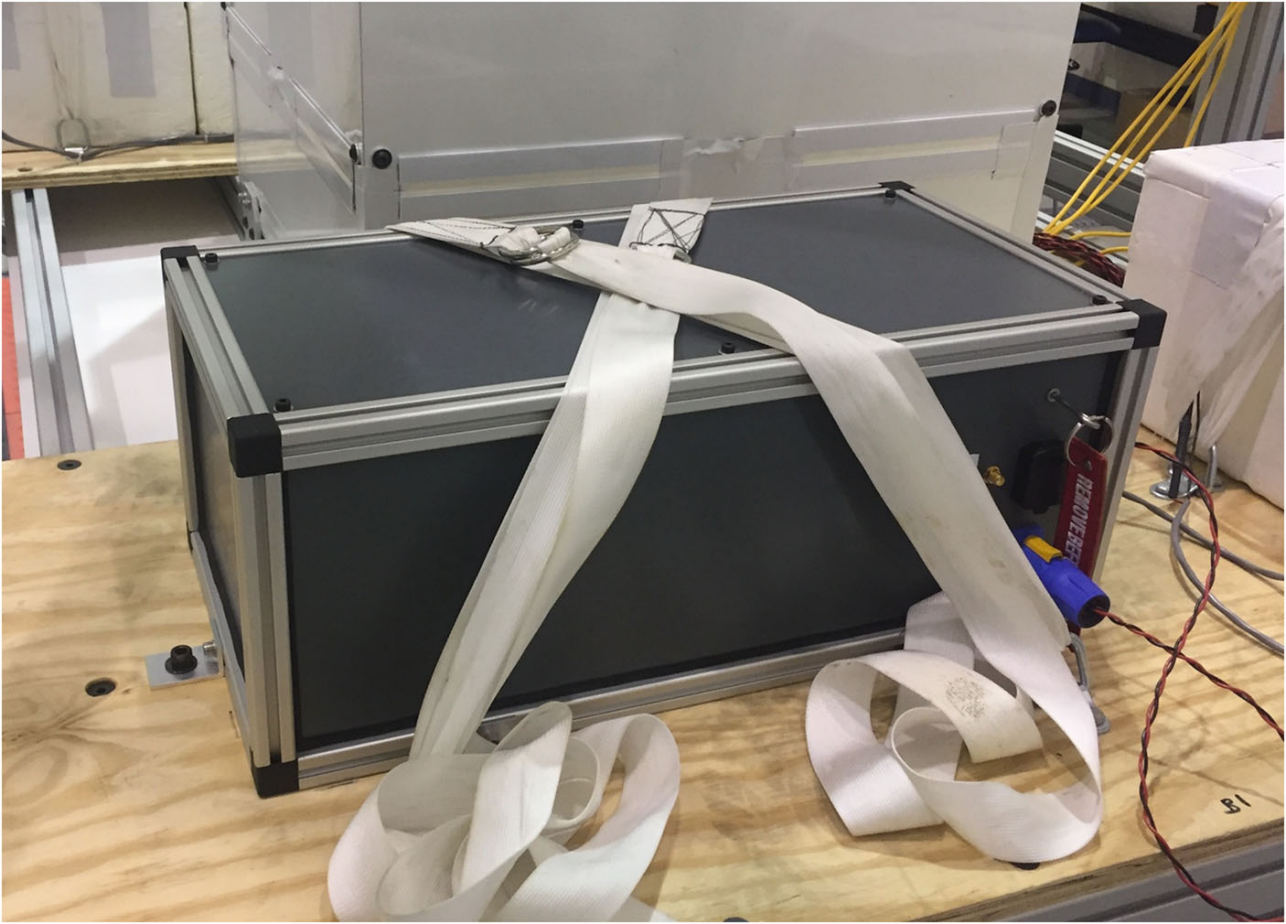
GMoDem bolted and strapped to the ASCOT gondola

The ASCOT gondola included two plywood decks, one either side of the detector, which were used to carry batteries and the GMoDem payload. The GMoDem enclosure was bolted to one of the plywood decks using six brackets. Additional security was provided using nylon straps attached to D-rings which were installed on the gondola deck next to GMoDem. This arrangement is shown in Fig. 5.

## Balloon flight

The launch campaign began in February 2018 with a review of GMoDem’s flight support requirements by CSBF. Over the following three months, the GMoDem hardware and flight software were finalised and a mission-readiness review was conducted by CSBF. The flight hardware assembly was shipped to University of New Hampshire, Durham, USA (UNH) by courier to be transported to CSBF along with ASCOT and its ground support equipment. GMoDem was picked up from University College Dublin, Ireland (UCD) on 16 May 2018 but, due to a customs delay upon entering the United States, did not arrive at UNH until 24 May.

The UCD team members arrived at CSBF on 3 June. Over the following days, GMoDem was carefully unpacked and check-out tests of the instrument were conducted. These tests included the pre-flight test and calibration of the gamma-ray instrument itself, testing of the housekeeping sub-systems including GPS and satellite communication, and ensuring that the PSU worked correctly with the CSBF batteries. Following satisfactory check-out of GMoDem and of the ASCOT instrument by the team from UNH, GMoDem was mechanically integrated into the ASCOT gondola. The fully integrated gondola then underwent a “hang” test and a flight-readiness review was conducted.

Unfortunately, the successful flight-readiness review was followed by several weeks of unsuitable weather conditions caused by a tropical storm forming in the Gulf of Mexico. Due to prior commitments, it was not possible for the UCD GMoDem team to remain at CSBF beyond 25 June. The UNH ASCOT team very kindly offered to prepare and, if necessary, deactivate GMoDem for the upcoming launch attempts while the UCD team remotely operated GMoDem via the Iridium satellite communication system.

**Fig. 6:**
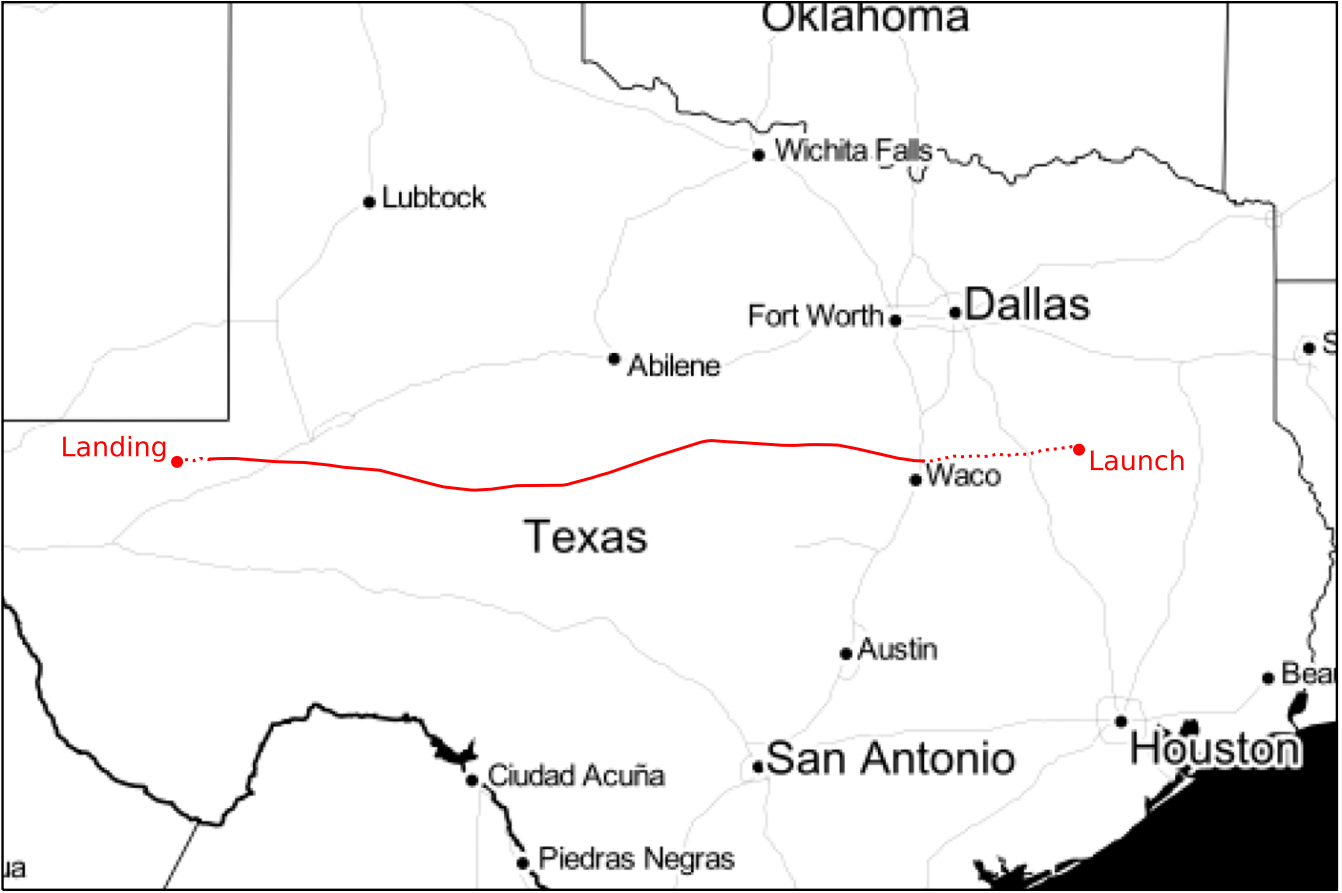
Map of the balloon flight path. The solid red section indicates where the balloon was at the float altitude of 37.4 km. The dotted sections indicate the ascent and descent phases of the flight

Following several launch attempts in early July cancelled due to unfavourable weather conditions, the balloon was successfully launched on the morning of 5 July 2018. A summary of the balloon flight is given in Table 1. Two hours after the launch the balloon reached its float altitude of 37 km. The float period lasted just over five hours, with the balloon travelling a distance of about 700 km from the launch site (Fig. 6). After that the ground crew sent the command to separate the gondola from the balloon terminating the flight. Deploying a parachute, the gondola experienced a rapid descent which lasted about 40 minutes. It landed near the New Mexico border, close to the town of Pecos where it was recovered the next day. The total duration of the flight was just over eight hours.

**Table 1:** ASCOT balloon flight summary

Flight Number:	1600 P
Date/Time Launched:	5 July, 2018 12:13:09 UTC
Launch Site:	Palestine, TX
Balloon Weight:	761 kg
Experiment Weight:	585 kg
Float Altitude:	37.4 km
Time Reached Float:	14:24:28 UTC
Termination Point:	31°42.73’ N, 103°12.43’ W
Termination Time:	19:32:54 UTC
Total Float Time:	05:08:26
Impact Position:	31°41.45’ N, 103°29.76’ W
Impact Time:	20:13:11 UTC
Total Flight Time:	08:00:02
Distance:	735 km

**Fig. 7:**
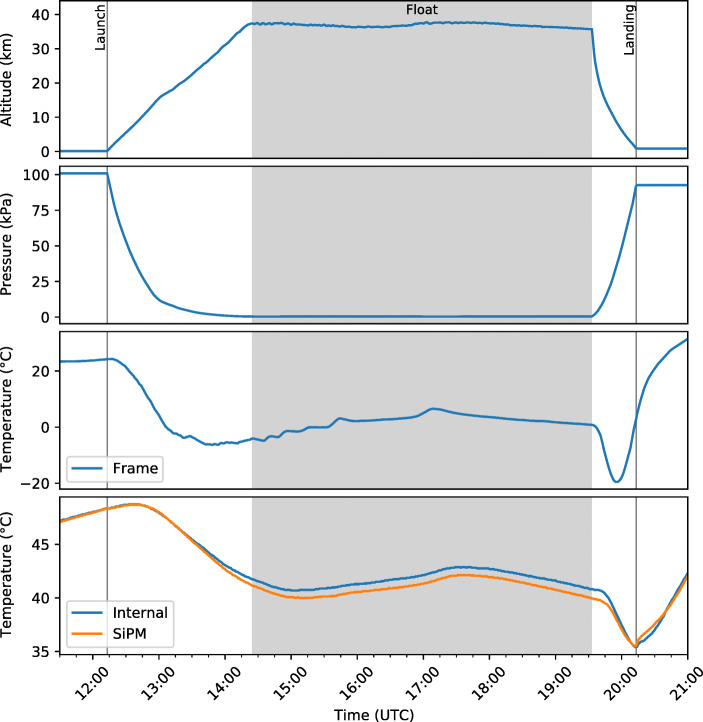
Logged housekeeping parameters as a function of time throughout the flight

**Fig. 8:**
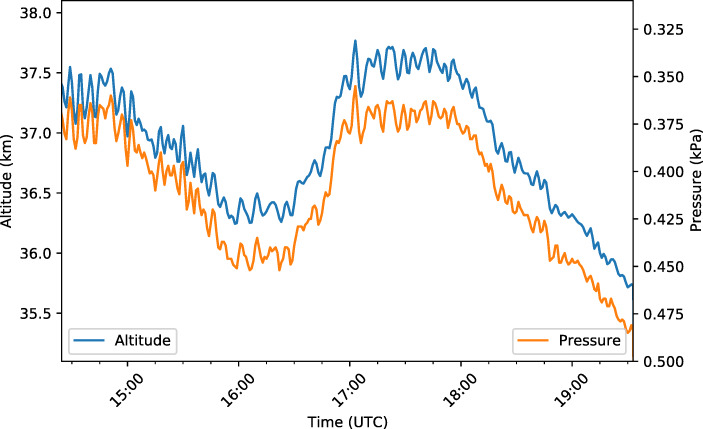
Altitude as logged by the u-blox M8 GNSS receiver and pressure as logged by the MS5611 barometric pressure sensor during the float period

GMoDem was powered up several hours before launch and the satellite telemetry allowed GMoDem to be monitored remotely to verify that it was functioning correctly and that all necessary software processes were running. The power was disconnected upon recovery of the payload, ending the acquisition of data. Figure 7 shows various parameters monitored throughout the experiment. We note that before launch the temperature inside the GMoDem enclosure reached approximately 48°C. This high temperature was mainly caused by relatively high power dissipated by the Galao board and OBC combined with good enclosure insulation and warm weather. After launch the internal temperature dropped by 8°C due to much colder environment at the float altitude and remained relatively stable during the float stage. As illustrated in Fig. 8, the pressure sensor was able to accurately record small changes in the atmospheric pressure tracing altitude variations during the float stage.

GMoDem was shipped back to Dublin about two weeks after the flight. Unfortunately upon its arrival it was discovered that the plastic housing of the scintillator had warped, with two of the sides bowing inwards and firmly clamping the scintillator inside the housing. The screws holding the housing to the SiPM array were found to be loose, also likely due the housing deformation as they had been well torqued before the balloon campaign. The housing deformation and loose attachment to the array may have caused an air gap or bubble between the scintillator and SiPMs. This problem can explain a sudden shift in the detector calibration observed between 2 July and 3 July (Section 5.1). The housing deformations is believed to have been caused by the high temperature inside the GMoDem enclosure rising to 51°C during the launch attempt on 2 July.

## Analysis

The data were analysed in Python using a variety of packages including Pandas, SciPy, NumPy, and Matplotlib. All data were imported to various Pandas data frames and indexed by time stamp allowing efficient binning based on time.

### SIPHRA data

The data generated by SIPHRA include a trigger-type flag, and the ADC values for each of the enabled channels. To record when each trigger has occurred, Galao prepends the SIPHRA data with the elapsed cycles of the SIPHRA clock. Correlation between the clock cycles and UTC is achieved in the flight software by inserting a standard UTC Unix time stamp into the recorded data after every 10 triggers.

The clock cycle count is represented as a 32-bit number by Galao and therefore rolls over at a value of 4,294,967,295. As the SIPHRA clock was run at 2.5 MHz, the clock count rolls over approximately once every 28 minutes and 38 seconds. The data are first processed by replacing the clock cycle count with a monotonically increasing clock cycle number which does not roll over. A linear relationship between the monotonically increasing clock count and the Unix time stamp can then be established which is used to reconstruct a Unix time stamp for each SIPHRA trigger.

Following time stamp reconstruction, the data are then separated by trigger type. The trigger-type flag can be used to determine if triggers are internally or externally generated. The internally generated triggers are pulses from the SiPMs corresponding to events or interactions of gamma rays in the scintillator while the externally generated triggers are forced readouts initiated by Galao at a fixed rate. These forced readouts are used to measure the baseline signal throughout the flight. Separate Pandas data frames are used to store the event data (internally generated triggers) and baseline data (externally generated triggers).

### Baseline subtraction

**Fig. 9:**
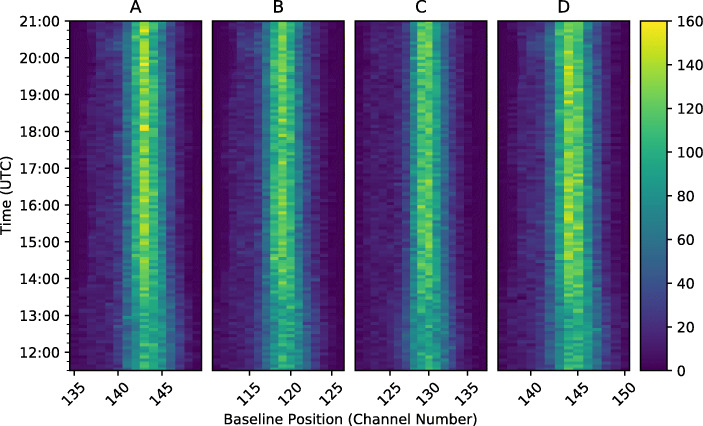
Five minute binned spectra of the baseline counts in the individual SiPM subarrays for the entire flight

The first step in processing the event data is to subtract from each pulse-height measurement the baseline value corresponding to the zero signal. The baselines for all SIPHRA channels are sampled during short dedicated runs using periodic externally forced readouts. In addition, external triggers are generated at a 2 Hz rate during normal data taking to determine if the baseline signal shows any evolution throughout the flight. The triggers are then sorted into bins each representing 5 minutes of time throughout the flight. Within these time-bins, the triggers are sorted into a histogram by ADC value to generate a spectrum of the baseline values. The baselines for the four SIPHRA channels connected to the SiPM subarrays (labeled A–D) are shown in Fig. 9.

As the external triggers are generated at a fixed rate, it is entirely possible to externally trigger SIPHRA when there is scintillation light incident on the SiPMs and therefore return a value that is not a true baseline value. These values are typically much larger than the true baseline values and so are easily removed by sigma clipping the baseline values at 5*σ*.

Once these outliers have been removed, the distributions within each 5 minute bin are fit to determine the baseline values for each bin. The baseline spectra are very well described by a Gaussian distribution.

### Response linearisation

The SiPM response is not strictly proportional to the number of incident photons. This non-proportionality becomes noticeable when the number of photons is large enough to fire a significant fraction of SiPM microcells (e.g. [28]). GMoDem also suffers from additional non-linearity caused by the SIPHRA ASIC which completely saturates at a level of about 3800 ADC channels (out of 4096 channels for the 12-bit ADC). To correct for SiPM and SIPHRA non-linearity, a set of four correction functions were developed, one for each subarray connected to a single SIPHRA input. These functions are applied to the data after baseline subtraction.

**Fig. 10:**
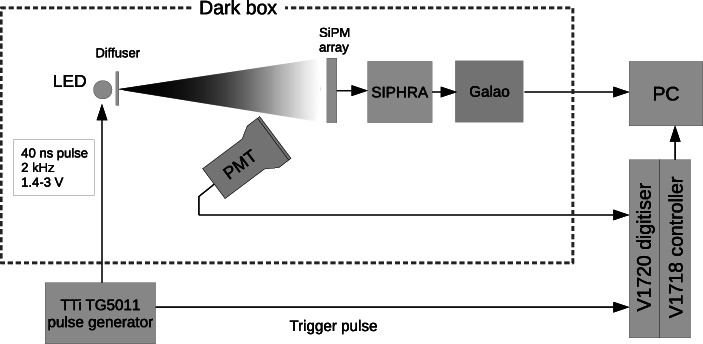
Schematic diagram of the set-up for SiPM non-linearity measurements

The correction functions are based on measurements of the response of GMoDem’s SiPM array to light pulses produced by a light emitting diode (LED). The measurements were performed in a light-tight box using a set-up shown in Fig. 10. The SiPM array was placed at a distance of 40 cm opposite a 430 nm blue LED which was equipped with a light diffuser to provide uniform illumination on the SiPM array surface. The LED was pulsed with 40 ns square pulses generated by an Aim-TTi TG5011 function generator with 2 kHz frequency. The light intensity was controlled by changing the pulse amplitude between 1.38 – 3.00 V. The SIPHRA ASIC and SiPM biasing was configured as during the balloon flight.

A Hamamatsu R6233-100 (3-inch, 8-stage) PMT was used for independent measurements of light intensity. The PMT was equipped with a tapered voltage divider to improve pulse linearity and biased to 1000 V. It was positioned such that the reflected light from the SiPM array was directed towards the PMT, thereby reducing the amount of light detected and preventing PMT response non-linearity. The PMT signal was sampled and digitally integrated using a CAEN V1720 waveform digitiser (12-bit, 250 MS/s).

**Fig. 11:**
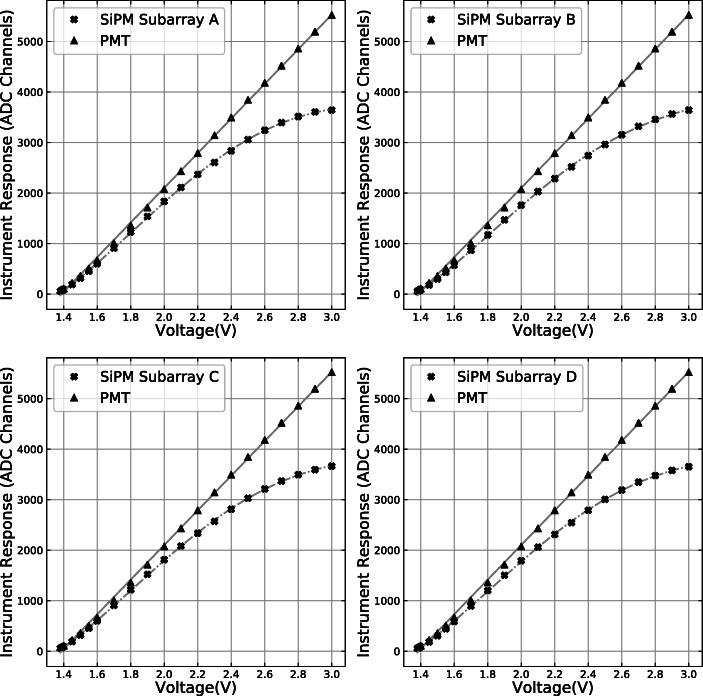
Signals from the PMT and four GMoDem SiPM subarrays measured as a function of LED pulse amplitude

**Fig. 12:**
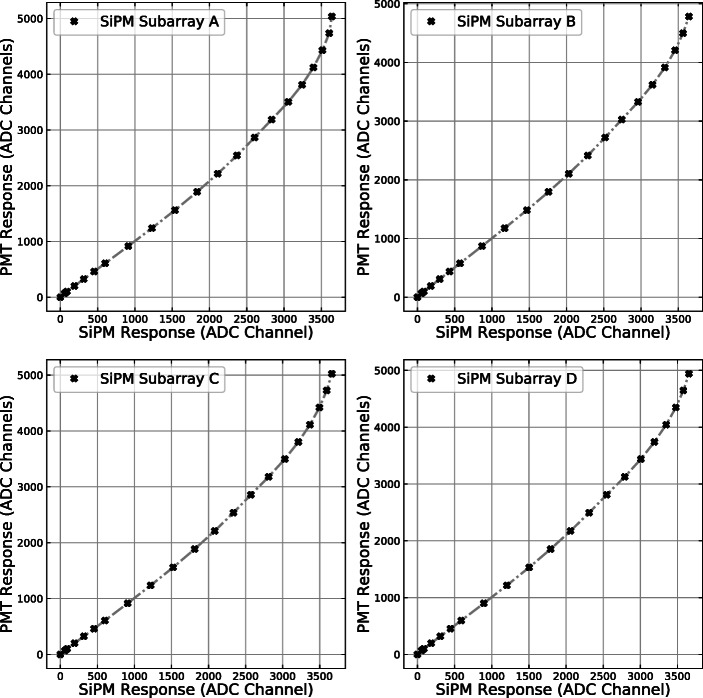
Re-scaled PMT signal as a function of the SiPM signal for each subarray. The response correction function is a cubic spline interpolation between the respective SiPM subarray and PMT response data

The SiPM and PMT responses were recorded in terms of digitised pulse height as shown in Fig. 11. The PMT demonstrated a nearly linear signal dependence on the LED pulse amplitude (voltage setting of the function generator), indicating that the number of photons emitted by the LED has an almost linear relationship with the pulse amplitude, although deviations from the linear behaviour can be seen below 2 V. At the same time, the SiPMs with SIPHRA readout showed apparent non-linearity and saturation for strong light pulses. By relating the signals from the four SiPM subarrays to the response of the PMT (assumed to be proportional to the number of emitted photons), linearisation corrections were generated across the range of ADC channels. A plot of the relationship between PMT and GMoDem SiPM response is shown in Fig. 12 for all four SiPM channels. The PMT response data has had a re-scale factor applied to give a near match of the SiPM response values. The re-scale factor was calculated by fitting a linear function to the relationship between the PMT and SiPM response in a low signal range (< 2000 channels for SiPM response). This re-scale factor is not particularly important, as although the PMT response is linear, the units of measurement are essentially arbitrary. For each SiPM subarray, the response correction function was generated by cubic spline interpolation between the SiPM subarray and PMT response data as shown in Fig. 12. Above 3600 ADC channels the GMoDem response becomes saturated and cannot be reliably corrected, as a small error in the measured SiPM response corresponds to a large change in the linearised signal. At this point it is only possible to determine the lower limit for the linearised signal.

The observed GMoDem SiPM non-linearity is a combined effect of the intrinsic SiPM non-linearity and the SIPHRA readout. Theoretical calculation of the SiPM non-linearity requires an accurate SiPM characterisation including such effects as microcell recovery, optical crosstalk and afterpulsing. When the light pulse is short relative to the microcell recovery time (∼50 ns for the SiPMs used in GMoDem), the SiPM response can be approximately estimated using a simple saturation model [26, 28]:

1 $$ N_{\text{fired}} = N_{\text{tot}}(1-e^{-N_{\text{pe}}/N_{\text{tot}}}),  $$

where *N*_fired_ is the average number of fired SiPM microcells (which defines the size of the SiPM signal), *N*_tot_ is the total number of microcells in the SiPM and *N*_pe_ is the number of photoelectrons produced by the light pulse. The number of photoelectrons can be estimated as

2 $$ N_{\text{pe}} = \frac{N_{\text{ph}} \text{PDE}}{1+\ln(1-\text{CT})},  $$

where *N*_ph_ is the number of incident photons, PDE is the photon detection efficiency and CT is the optical crosstalk probability. Equation (2) treats the crosstalk effect as a branching Poisson process and accounts for multiple microcells that may be fired in the proximity of a primary Geiger breakdown [35]. However, it neglects the effect of SiPM saturation on the development of the crosstalk process and therefore overestimates the number of photoelectrons produced in a saturated SiPM.

The exact relationship between the number of fired microcells and the ADC channel number for the GMoDem detector is not known, but it can be roughly estimated from the detector response to gamma rays. A typical light yield of an encapsulated CeBr_3_ crystal is about 40 ph/keV [24], which gives 26480 photons for a 662 keV gamma ray fully absorbed by the crystal, or 11383 photoelectrons assuming PDE = 0.37 for CeBr_3_ emission (*λ*_peak_ = 380 nm) and CT = 0.13 [22]. Comparing the number of photoelectrons to a mean observed signal of 2018 linearised ADC channels from a fully absorbed 662 keV gamma ray (Section 5.1), the conversion factor is estimated to be around 5.6 photoelectrons per channel. The maximum signal of an SiPM subarray in Fig. 12 is about 5000 linearised ADC channels, which corresponds to 28000 photoelectrons. Each 4-SiPM subarray has 89168 microcells in total, therefore Equation 1 predicts 24030 fired microcells for 28000 photoelectrons. The ratio *N*_fired_/*N*_pe_ = 0.86 indicates a 14% deviation from the linear response, as *N*_fired_ ≈ *N*_pe_ in a low-intensity linear range (*N*_pe_ ≪ *N*_tot_). The SiPMs can thus be expected to have a moderate non-linearity up to about 14% over the full ADC range. The SIPHRA readout, however, results in much stronger non-linearity for large signals and completely saturates at about 3600 ADC channels.

The correction functions described above aim to correct the non-linear effects in the SiPMs and SIPHRA readout. It should be noted, however, that the SiPM response also depends on the SiPM overvoltage (excess bias voltage above the breakdown voltage). Therefore, linearisation functions are only valid for a specific overvoltage; it was essential that the non-linearity measurements were performed at the same overvolatge as used during the balloon flight. In addition, the SiPM response non-linearity depends on the duration of the light pulse: it decreases for long pulses that are comparable or longer than the SiPM recovery time. The scintillation decay time constant for CeBr_3_ is about 20 ns [24], which is smaller than the 40 ns pulse duration used in the LED measurements. This means that the correction functions generated from the LED measurements may undercorrect the non-linearity of the GMoDem detector. This effect is not expected to be large, as in both cases the light pulse duration is smaller than the SiPM recovery time (50 ns specified by the manufacturer but possibly longer as the recovery time can be affected by the readout impedance).

No correction for scintillator non-proportionality is used in this work. Although this is an important effect for low energy gamma rays, the non-proportionality of CeBr_3_ scintillator does not exceed 5% for energies above 100 keV [24].

For each processed event, the correction functions are individually applied to the baseline-subtracted signals from the four subarrays A–D. The total detector signal is then calculated by adding the four linearised signals.

## Results

### Pre-flight calibration and performance

**Fig. 13:**
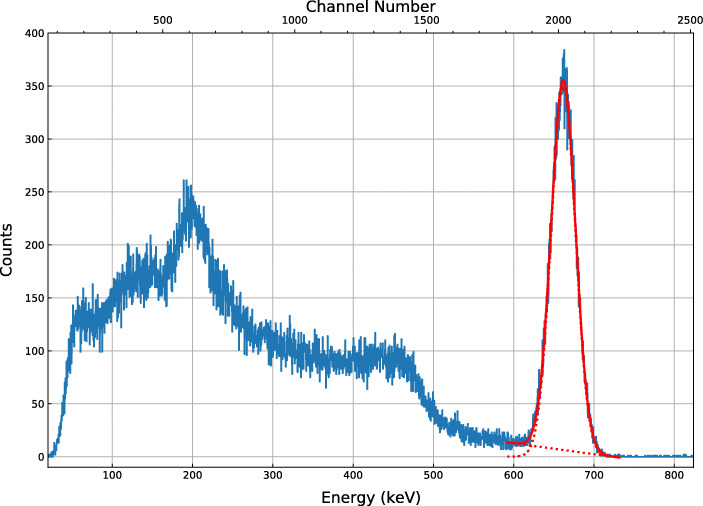
Spectrum of ^137^Cs measured by GMoDem

A pre-flight calibration of the detector was conducted on 3-4 June 2018 (one month prior to the eventual launch of GMoDem) using gamma rays from ^22^Na and ^137^Cs radioisotopes. The baseline-subtracted and linearised spectrum recorded for the ^137^Cs source is shown in Fig. 13. A sum of a Gaussian and a linear function is used to fit the full-absorption peak for 662 keV gamma rays emitted by the source and a contribution from background and incompletely absorbed events. The ull width half maximum (FWHM) of the Gaussian characterises the energy resolution of the detector and was found to be 5.47% . The peak positions and widths for all measured gamma-ray lines are listed in Table 2. The measured line positions establish the energy scale factor of linearised ADC channels: 0.328 keV/channel.

**Table 2:** Positions and widths of the different gamma-ray lines measured one month before the flight

Energy	Isotope	Mean	FWHM
(keV)		(ADC channel)	(%)
511	^22^Na	1558.96 ± 0.07	6.2 ± 0.02
662	^137^Cs	2018.35 ± 0.25	5.47 ± 0.02
1274	^22^Na	3883.02 ± 0.24	4.08 ± 0.02

**Fig. 14:**
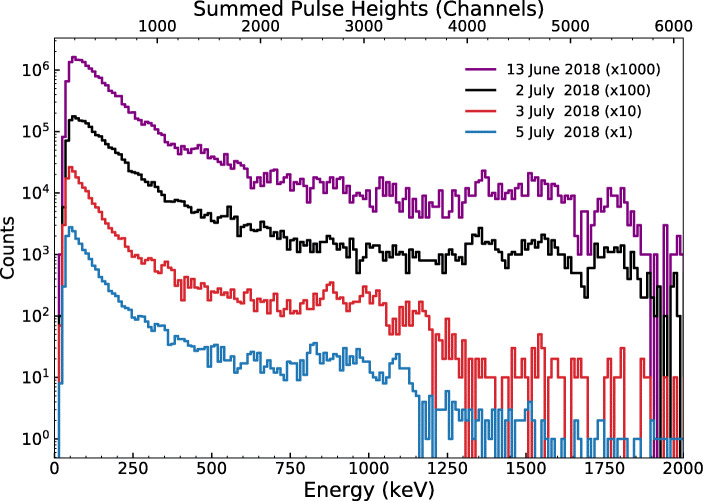
Background spectra taken by GMoDem on different dates using an acquisition time of 30 minutes. For visual separation the counts are multiplied by different scale factors given in the legend. All spectra were measured under similar temperature conditions, with the SiPM temperature rising during the measurements from 30°C to 36°C. The energy scale is from the calibration performed on 3 June 2018

Background spectra measured with the GMoDem detector (Fig. 14) feature three characteristic peaks at approximately 1.35 MeV, 1.55 MeV, and 1.8 MeV due to intrinsic *α* emissions from the decay products of ^227^Ac naturally present in the CeBr_3_ crystal [25]. Because of the low count rate, these peaks are not suitable for an accurate detector calibration but they can be used to track significant changes in the detector energy scale. As shown in Fig. 14, the energy scale was stable between 13 June and 2 July. However, the detector signal dropped approximately by a factor of 1.6 between 2 July and 3 July and further reduced by launch on 5 July, rendering the pre-flight calibration invalid for in-flight measurements. This sudden reduction in the detector signal is associated with the damage to the plastic detector housing discovered after the flight which probably resulted in poor optical coupling between the scintillator and SiPMs. After GMoDem was shipped back to UCD and the scintillator was recoupled to the SiPM array, the detector response to 511 keV gamma rays from a ^22^Na source was found to be consistent with the pre-flight calibration.

The above-mentioned change in the detector signal also affected the energy range of the detector. SIPHRA measurements are triggered by pulse current exceeding a set threshold. The effective gamma-ray energy threshold (corresponding to the low energy spectral cut-off in Fig. 13) was initially about 50 keV but increased to 80 – 100 keV after the calibration shift. The detector energy range thus changed from approximately 50 keV – 4.5 MeV to 100 keV – 8.5 MeV.

### Light curve

**Fig. 15:**
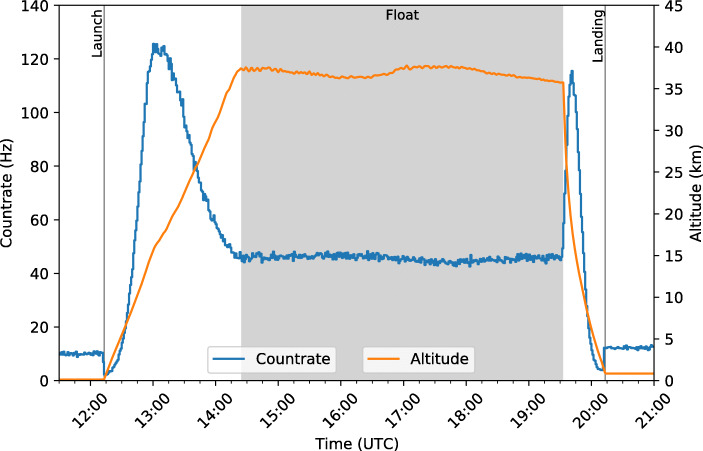
Light curve for the entire flight indicating the terrestrial background radiation detected at the launch and landing sites, the Pfotzer maxima detected during ascent and descent, and the relatively stable count rate experienced throughout the float period

For the purposes of generating a light curve, the event data were simply sorted into 1 minute bins to determine the count rate throughout the flight. The flight light curve is shown in Fig. 15 with the launch and landing times and the float period indicated.

The balloon was launched at 12:13 UTC and it can clearly be seen in the light curve that prior to launch, there is an initial background rate of 10 Hz detected at CSBF. Following launch, this background rate rapidly decreases with increasing altitude reaching a minimum of 2.2 Hz before atmospheric radiation effects cause the count rate to increase. As the balloon ascends, the Pfotzer maximum, the point of maximum radiation intensity in the atmosphere as a function of altitude, can clearly be seen in the light curve. This maximum count rate of 125.6 Hz was detected at approximately 13:00 UTC, at an altitude of approximately 16 km.

Following the maximum, the count rate decreases until it stabilises as the balloon achieves its float altitude of approximately 37.4 km. The float period beginning at 14:24 UTC is seen to have a relatively stable count rate which is expected due to the stability of the float altitude as seen in Fig. 7. The float count rate does vary slightly with the variations in altitude between approximately 44–47 Hz.

The balloon flight was terminated at 19:32 UTC and the descent demonstrates similar features to the ascent though on a shorter timescale due to the descent velocity being greater than that of ascent. An increase in the count rate is once again observed due to passage through the Pfotzer maximum region of the atmosphere at approximately 19:45 UTC, achieving a maximum count rate of 115.5 Hz. As the atmospheric radiation decreases, a minimum count rate of 3.9 Hz is detected before the count rate increases again to 12.2 Hz due to to ground-level background radiation when the balloon lands at 20:13 UTC.

### Baselines

**Fig. 16:**
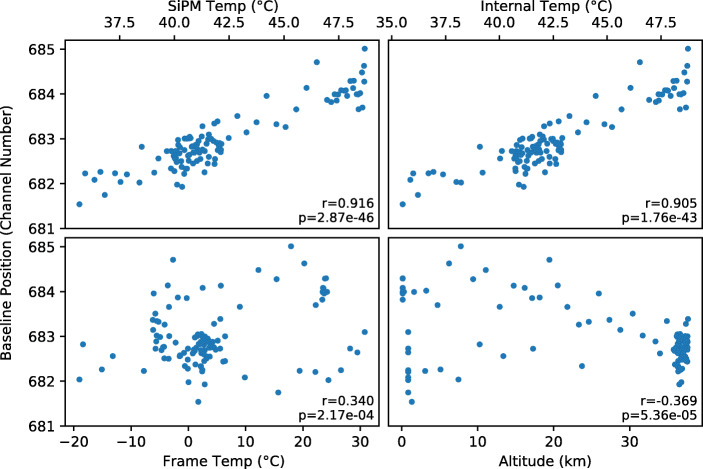
Correlation between the baseline position and various logged housekeeping parameters. The correlation coefficients and corresponding p-values are printed for each dataset

The signal baselines for all four SIPHRA channels showed small variations throughout the flight limited to a fraction of one ADC channel. To investigate the source of these variations, a sum of the four baselines (which essentially represents the combined baseline for the total detector signal) was compared to the recorded housekeeping data. Figure 16 shows correlation plots and values between the combined baseline position and the SiPM array temperature, the GMoDem enclosure internal temperature, the frame temperature, and the altitude.

**Fig. 17:**
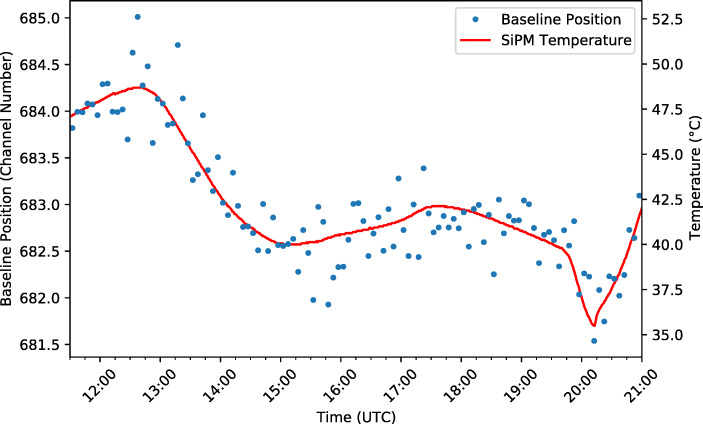
Baseline position throughout the flight overplot with the temperature inside the GMoDem enclosure demonstrating the correlation between the two parameters

A strong positive correlation was found between the baseline position and temperatures recorded within the GMoDem enclosure. The best correlation of 0.916 was found for the SiPM temperature, with the enclosure internal temperature also providing a good correlation of 0.905. Based on a linear fit between the parameters, Fig. 17 shows the SiPM temperature overplot on the baseline position as a function of time. The average shift for the combined baseline amounts to 0.19 channel/°C.

### Spectra

For processing into spectral data products, the event data were sorted into bins representing 15 minutes of time throughout the flight. The baseline values were calculated for each time bin.

**Fig. 18:**
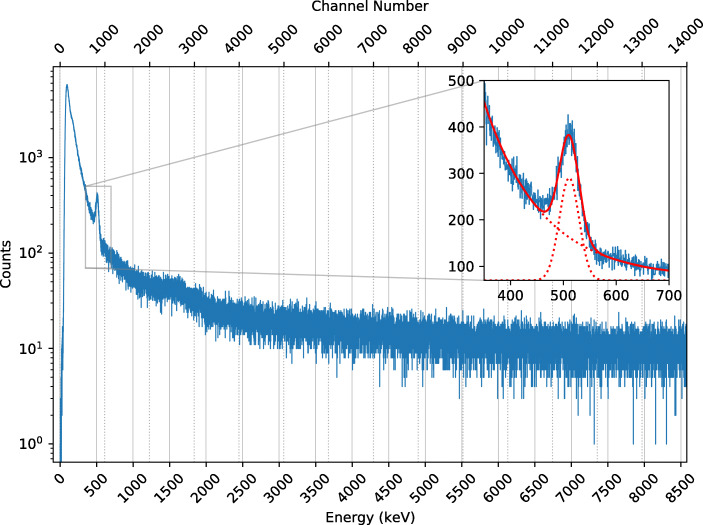
Recorded spectrum for the entire flight after baseline subtraction and linearisation. The low-energy cut-off corresponds to a trigger threshold of about 100 keV. A fit to the 511 keV is shown in inset. The overall fit is indicated in red, with the constituent components of the fit model shown as dotted lines

The baseline-subtracted and linearised spectrum for the entire flight is shown in Fig. 18. A notable feature of this flight spectrum is the detection of the 511 keV annihilation line at a detector channel value of approximately 835. This value is 1.87 times lower than that measured for the 511 keV line in the pre-flight calibration performed at CSBF which is explained by the signal drop discussed in Section 5.1 and a temperature effect discussed in Section 5.4.2.

As the pre-flight calibration cannot be used, the flight spectrum displayed in Fig. 18 has been calibrated using the 511 keV line detected in flight. Due to the larger background, the *α*-emission from the ^227^Ac decay products is barely visible in the spectrum as a small bump around 1.5–2 MeV. The position of this feature in the calibrated spectrum very roughly confirms that the energy calibration is valid and the detected line corresponds to 511 keV.

#### Time-resolved spectrum

**Fig. 19:**
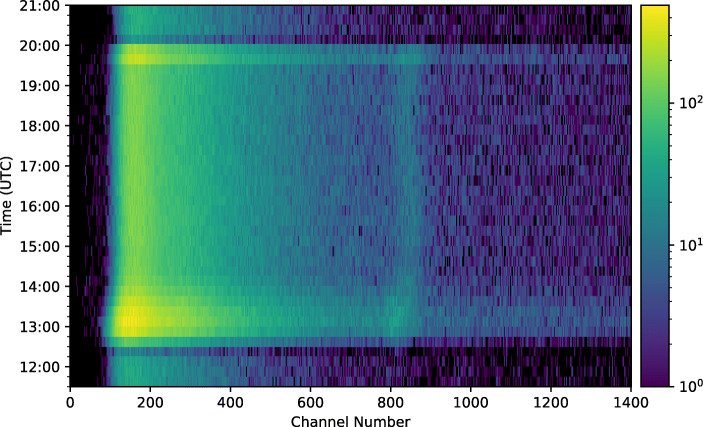
Time-resolved spectrum for the entire flight after baseline-subtraction and linearisation. The data have been binned into 15 minute intervals

A time-resolved spectrum based on the aforementioned 15 minute bins is shown in Fig. 19. This plot reveals many features which are also evident from the light-curve and full flight spectrum such as the Pfotzer maxima which are seen as bright horizontal bands across the plot at 13:00 and 19:45 UTC, the decrease in count rate, seen as dark horizontal bands following launch at 12:13 UTC, and prior to landing at 20:13 UTC, and the relatively stable count rate for the duration of the float period. The detection of the 511 keV line can be seen as a bright vertical band, once again at approximately a channel number of 835.

It can clearly be seen that the position of the 511 keV line in Fig. 19 is not stable, but in fact moves throughout the flight. As this peak originates from detection of gamma-ray photons with a known energy of 511 keV, in a well-calibrated instrument the position of this line should remain constant. Unfortunately, the overall spectrum is rather featureless and therefore the 511 keV line provides the only reference point that can be used to determine any shifts or variations in the spectrum. It is assumed that the variation in the position of this line represents an overall variation in the detector calibration or gain.

#### 511 keV line shift

**Fig. 20:**
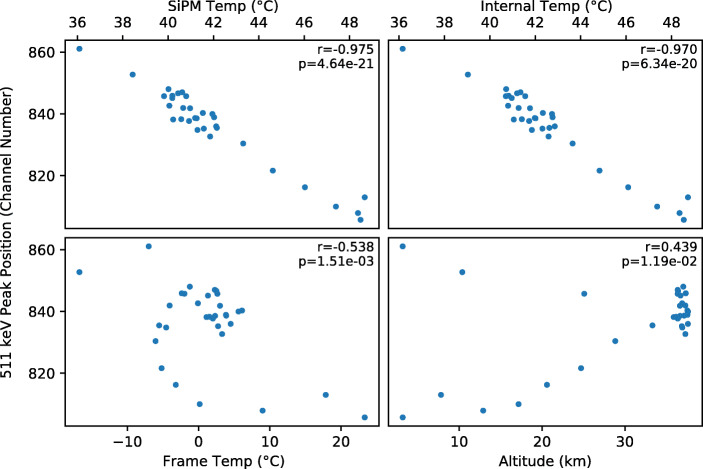
Correlation between the 511 keV peak position and various logged housekeeping parameters. The correlation coefficients and corresponding p-values are printed for each dataset

**Fig. 21:**
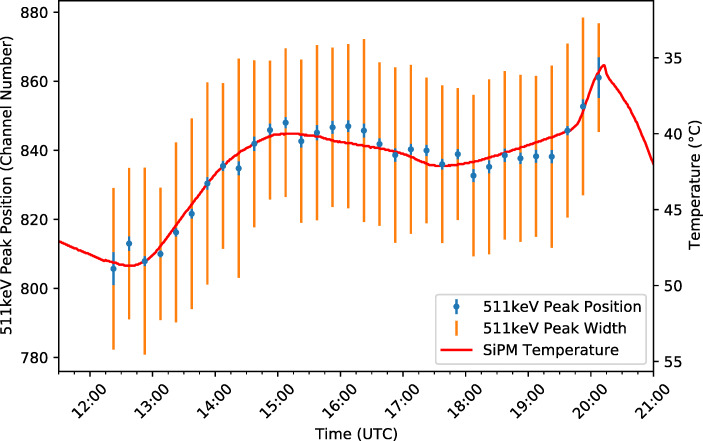
Position of the 511 keV line throughout the flight overplot with the SiPM array temperature demonstrating the strong correlation between the two parameters

To investigate the source of this variation, the 511 keV line position was compared to the recorded SiPM array temperature, the GMoDem enclosure internal temperature, the frame temperature, atmospheric pressure, and the altitude (Fig. 20). A very strong negative correlation was found between the detector gain and temperatures recorded within the GMoDem enclosure. The best correlation of -0.975 is found for the SiPM temperature, with the enclosure internal temperature also providing a good correlation of -0.970. Based on a linear fit between the parameters, Fig. 21 shows the SiPM temperature overplot on the 511 keV line position as a function of time. The 511 keV line is shifted by -4.3 channel/°C which corresponds to a relative change of -0.5%/°C.

The detector gain is seen to have a negative correlation with SiPM temperature. As the temperature increases, the gain of the detector decreases and so too does the position of the 511 keV line. This behaviour can be well explained by the 21.5 mV/°C temperature dependence of the breakdown voltage of the J-series SiPMs. As the temperature increases, the breakdown voltage of the SiPMs also increases. For a fixed bias voltage, an increased breakdown voltage gives a decreased overvoltage and therefore a reduced SiPM gain.

As detailed in Section 2.5, GMoDem was designed with an adjustable power supply specifically for the purpose of adjusting the bias voltage in order to maintain a constant overvoltage and therefore constant gain, compensating for variations in the temperature of the SiPMs. During the flight, the GMoDem flight software was constantly adjusting the bias voltage of the SiPM array based on the SiPM array temperature in order to correct for the breakdown voltage temperature dependence and was programmed to do so at a rate of 21.5 mV/°C. The negative correlation between temperature and gain which is obtained from the flight data suggests that the bias power supply was under-correcting for the expected temperature dependence. Post-flight investigations revealed that the GMoDem PSU itself was sensitive to temperature and at higher temperatures the generated bias voltage was below the programmed value, which is believed to be origin of the observed effect.

#### Scale correction

Accepting that the bias power supply in GMoDem under-performed during the flight, it is possible to consider the 511 keV line as a fixed point that should have remained constant and therefore determine a detector calibration as a function of time throughout the flight. This effectively corrects for the incorrect bias voltage that was applied during the flight.

**Fig. 22:**
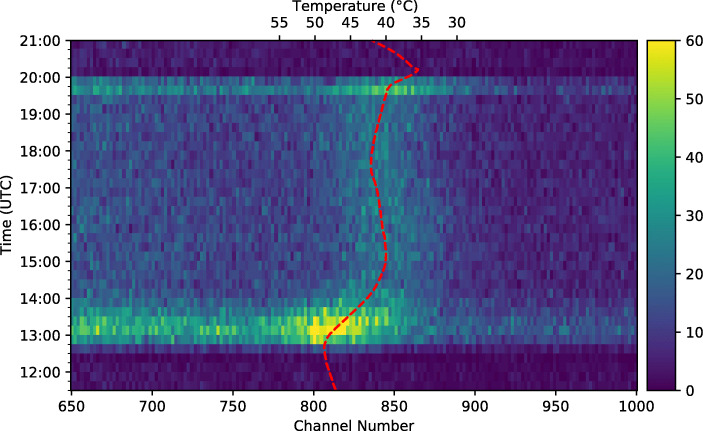
The SiPM array temperature overplot on the time-resolved spectrum of the 511 keV peak, clearly demonstrating the relationship between SiPM temperature and gain, and the inability of the GMoDem PSU to compensate the change in SiPM breakdown voltage

The first approach to doing so parametrises the detector scale factor as a function of the SiPM array temperature utilising the linear fit between the temperature and the 511 keV position. Figure 22 shows a time-resolved spectrum of the 511 keV line overplot with the SiPM array temperature having been transformed to channel-space according to the fit between temperature and the 511 keV line position shown in Fig. 21. Assuming that the temperature curve approximates that correct channel number position of the 511 keV line at any given time, it is possible to scale every individual event based on the logged SiPM temperature.

The second, simpler approach to correcting for the incorrect applied bias voltage is to determine a scale factor for each of the 15-minute time bins and then to apply this scale factor to all events within the bin. The scale factor is determined by considering the fit position of the 511 keV line in each time bin and comparing to the mean position of the line during the float period.

**Fig. 23:**
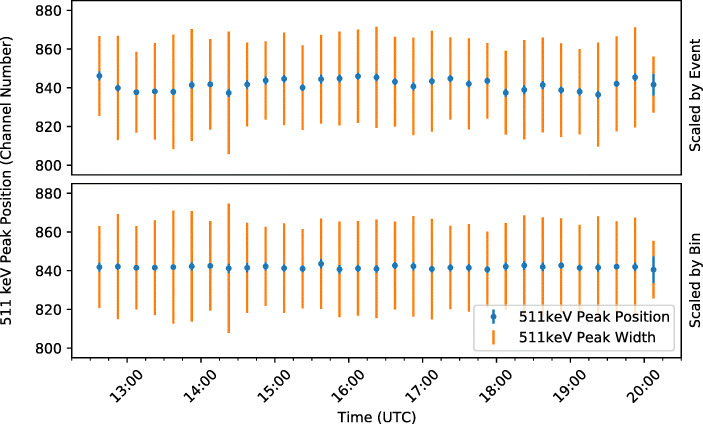
Position of the 511 keV line throughout the flight after individual scaling of each of the event values according to temperature (top) and after group scaling of all events within each time bin to align the 511 keV peaks (bottom)

The resulting position of the 511 keV line after scale corrections is shown in Fig. 23 for both methods. Either method works well and results in a relatively stable position of the line throughout the flight. The advantage of the second method is that it can correct any changes in the detector energy scale whether they are temperature related or not. However, it requires a constant observation of the 511 keV line (or another calibration source) and also needs relatively long time bins to accumulate a sufficient number of events. Therefore unlike the first method, it cannot properly correct fast changes in the detector scale.

We also note that the second method deceivingly corrects for statistical fluctuations of the mean position of the 511 keV line in each time which explains the nearly constant line position in the bottom plot in Fig. 23. This effect would not have been observed with an independent sample. Small variations are still present in the plot, because the scale corrections affected the event binning into a spectrum and the range of events used for the 511 keV line fit.

The temperature dependence of the detector signal and the resulting variations of the 511 keV line position contribute to the observed width of the 511 keV peak and thereby degrade the energy resolution of the detector. The scale correction methods correct for this effect, therefore they are expected to reduce the width of the 511 keV peak. The width of the peak obtained with and without corrections is given in Table 3 for different time periods of the flight. Indeed, both correction methods reduce the width of the peak. The effect is most notable when considered over the full flight, accounting for the large temperature change which occurred during the flight.

**Table 3:** Width of the 511 keV peak at various periods throughout the flight. The peak width is given as recorded by GMoDem and after both bias voltage correction methods have been applied

	Start	End	Peak FWHM (%)		
			Recorded	Scale Event	Scale Bin
Pfotzer Max (Up)	12:30	14:00	7.31	7.19	7.11
Float All	14:15	19:30	6.73	6.64	6.65
Float Hour 1	14:15	15:15	6.82	6.72	6.78
Float Hour 2	15:15	16:15	6.59	6.54	6.55
Float Hour 3	16:15	17:15	6.91	6.86	6.87
Float Hour 4	17:15	18:15	6.07	6.11	5.98
Float Hour 5	18:15	19:15	6.63	6.72	6.70
Pfotzer Max (Down)	19:30	20:00	7.01	6.87	6.69
Total	12:30	20:00	7.83	6.83	6.78

The width of 6.8% observed for the 511 keV peak during the flight is larger than 6.2% measured in the pre-flight calibration using a ^22^Na source, which can again be explained by the damaged detector housing and poor optical coupling between the scintillator and SiPMs.

## Conclusions and future work

Due to the relatively short time of about three months between the start of the project and the balloon flight opportunity, GMoDem was constructed as a simplified prototype detector using commercially available components: a CeBr_3_ crystal, SiPM arrays, a SIPHRA evaluation board, and a Galao interface board. A 3D-printed plastic housing was used to attach the scintillator to the SiPMs and seal the detector from ambient light.

The detector demonstrated continuous operation during the 8-hour balloon flight including the 5-hour float at 37-km altitude and after the hard landing. It performed spectral measurements in an energy range of 100 keV to 8 MeV and observed the 511 keV gamma-ray line arising from positron annihilation in the atmosphere with full width at half maximum of about 7%. During the ascent and descent stage, the detector count rate peaked at an altitude of approximately 16 km corresponding to the point of maximum radiation intensity in the atmosphere.

The performance of the detector was affected by two problems discovered during the data analysis and post-flight examination. Prior to launch, the plastic housing of the detector was significantly damaged by the relatively high temperature inside the payload enclosure, which resulted in poor coupling of the scintillator and SiPMs. This reduced the detector signal by a factor of 1.6 and caused a loss in the energy resolution. The second problem was a significant dependence of the detector signal on temperature. It is attributed to a temperature dependent deviation of the SiPM bias voltage generated by the PSU from the requested value. This is a power supply issue that can be remedied by using a voltage monitor for the generated bias voltage. It is also possible to track and correct for variations in the signal scale using the position of the 511 keV line in a time-resolved spectrum.

The balloon test did not reveal any other problems or concerns for detector operation in space. The results of this study confirm the feasibility of using CeBr_3_ scintillator, SiPMs, and SIPHRA readout circuit in future space missions.

The effects of SiPM radiation damage on the detector performance have been assessed in a follow-up study, where the same SiPMs were irradiated with 101.4 MeV protons [33]. Depending on orbit and mission lifetime, the SiPM dark current and noise can increase by orders of magnitude, increasing the power requirements and limiting the ability of the detector to detect low energy gamma rays. However, a configuration using a bright CeBr_3_ scintillator together with a relatively small array of 16 SiPMs should still be able to detect 60 keV gamma rays after 6 years in a 550 km orbit with 40° inclination.

The same technology will be employed for the GMOD detector for the EIRSAT-1 satellite being built at UCD [21]. The main difference is that GMOD needs to be more robust and compact enough to fit into the 2U CubeSat. It will use a longer CeBr_3_ crystal and a custom-built 16-SiPM array composed of the same J-Series sensors. It will also have a small custom SIPHRA board adapted to the size of the detector. To reduce the power requirements of the detector, the Galao board based on the Zynq-7000 system on chip will be replaced by a low-power microcontroller solution. The detector housing will be made of aluminium, therefore it will not be prone to thermal damage observed in the balloon experiment. GMOD will also include a monitor for the SiPM bias voltage to prevent any discrepancy between the programmed voltage and the actual output.
